# Cytoreduction and HIPEC in The Netherlands: Nationwide Long-term Outcome Following the Dutch Protocol

**DOI:** 10.1245/s10434-013-3145-9

**Published:** 2013-07-30

**Authors:** Anke M. J. Kuijpers, Boj Mirck, Arend G. J. Aalbers, Simon W. Nienhuijs, Ignace H. J. T. de Hingh, Martinus J. Wiezer, Bert van Ramshorst, Robert J. van Ginkel, Klaas Havenga, Andreas J. Bremers, Johannes H. W. de Wilt, Elisabeth A. te Velde, Vic J. Verwaal

**Affiliations:** 1 Department of Surgical Oncology, The Netherlands Cancer Institute, Antoni van Leeuwenhoek Hospital, Amsterdam, The Netherlands; 2 Department of Surgery, Catharina Hospital Eindhoven, Eindhoven, The Netherlands; 3 Department of Surgery, Sint Antonius Hospital Nieuwegein, Nieuwegein, The Netherlands; 4 Department of Surgery, University Medical Center Groningen, Groningen, The Netherlands; 5 Department of Surgery, Radboud University Nijmegen Medical Center, Nijmegen, The Netherlands; 6 Department of Surgery, VU Medical Centre Amsterdam, Amsterdam, The Netherlands

## Abstract

**Purpose:**

This nationwide study evaluated results of cytoreductive surgery (CRS) combined with hyperthermic intraperitoneal chemotherapy (HIPEC) for peritoneal metastasis of colorectal origin in the Netherlands following a national protocol.

**Methods:**

In a multi-institutional study prospective databases of patients with peritoneal carcinomatosis (PC) from colorectal cancer and pseudomyxoma peritonei (PMP) treated according to the Dutch HIPEC protocol, a uniform approach for the CRS and HIPEC treatment, were reviewed. Primary end point was overall survival and secondary end points were surgical outcome and progression-free survival.

**Results:**

Nine-hundred sixty patients were included; 660 patients (69 %) were affected by PC of colorectal carcinoma and the remaining suffered from PMP (31 %). In 767 procedures (80 %), macroscopic complete cytoreduction was achieved. Three-hundred and thirty one patients had grade III–V complications (34 %). Thirty-two patients died perioperatively (3 %). Median length of hospital stay was 16 days (range 0–166 days). Median follow-up period was 41 months (95 % confidence interval (CI), 36–46 months). Median progression-free survival was 15 months (95 % CI 13–17 months) for CRC patients and 53 months (95 % CI 40–66 months) for PMP patients. Overall median survival was 33 (95 % CI 28–38 months) months for CRC patients and 130 months (95 % CI 98–162 months) for PMP patients. Three- and five-year survival rates were 46 and 31 % respectively in case of CRC patients and 77 and 65 % respectively in case of PMP patients.

**Conclusions:**

The results underline the safety and efficacy of cytoreduction and HIPEC for PC from CRC and PMP. It is assumed the uniform Dutch HIPEC protocol was beneficial.

Cytoreductive surgery (CRS) and hyperthermic intraperitoneal chemotherapy (HIPEC) as a treatment for peritoneal surface malignancies remains the subject to debate, especially amongst nonexperts. It was questioned whether there is enough scientific evidence for this high-risk procedure (New York Times, August 11, 2011). In the Netherlands, the outcome of this treatment was evaluated by a randomized, controlled trial published in 2003, which showed survival benefit for patients treated with CRS–HIPEC compared to chemotherapy and palliative surgery in an intention to treat analysis.^1^ Results of this trial were obtained in a single-expert centre. Because the benefits of the treatment were established, CRS–HIPEC procedures became widely available in now six hospitals in the Netherlands.

During the past decades, the incidence of colorectal carcinoma (CRC) has increased worldwide. Colorectal carcinoma is the second most common cancer in the Netherlands with an incidence of 12,000 new cases per year. In 2010, the colorectal mortality was 12 % of all cancer death in the Netherlands, with most deaths due to metastatic disease.^2^ Besides liver metastases, peritoneal metastases are a common sign of tumour progression or recurrence of colorectal cancer. Two population-based studies reported recently incidences of synchronous peritoneal metastasis of 4.3 and 4.8 %. Metachronous metastasis was reported to a rate of 4.2 %.^3^,^4^

Peritoneal metastases are generally associated with a poor prognosis. In a multicentre, prospective study, published in the year 2000, median overall survival was 5.2 months for patients with colorectal cancer, and all patients with this condition had a fatal outcome.^5^ Few studies have been performed to study the effect of chemotherapy on peritoneal metastasis of colorectal cancer. A median survival of 6 months was reported of patients with PC primarily treated with 5-FU and leucovorin.^6^ For patients with PC as the only metastatic site treated with systemic chemotherapy and palliative surgery somewhat better results were reported with a median overall survival of 12.6 months.^1^,^7^ In recently published subanalyses of chemotherapy studies, patients with peritoneal carcinomatosis had a poorer prognosis than patients with other metastatic sites, independent of the chemotherapy regimen.^8^,^9^

Because systemic chemotherapy has not been very efficient to treat intra-abdominal tumor dissemination, novel therapies were developed.^10^ CRS–HIPEC is now becoming the preferred treatment option for many peritoneal surface malignancies, such as peritoneal metastasis of colorectal cancer, malignant peritoneal mesothelioma, and pseudomyxoma peritonei (PMP).^11^ This treatment consists of local disease control by macroscopic complete cytoreductive surgery combined with hyperthermic intraperitoneal chemotherapy to remove microscopic residual disease completely.

The CRS–HIPEC treatment can improve survival of patients with peritoneal metastasis of colorectal cancer. Five-year survival rates of between 19 and 51 % have been reported.^12^–^14^ For PMP patients, 5-year survival rates of 73–86 % have been reported.^14^,^15^ Throughout the world, the CRS–HIPEC procedure is adopted as a possible curative procedure for peritoneal surface malignancies. However, there are many different treatment regimens for cytoreduction and HIPEC. The timing of the intraperitoneal chemotherapy is not universal. Possible treatment schedules include intraoperative intraperitoneal chemotherapy, early postoperative intraperitoneal chemotherapy (EPIC), or both. Also, several different chemotherapeutic drugs are used and applied in different doses and regimens.^15^,^16^

The question remains whether CRS–HIPEC once performed on a wider scale is beneficial. In this study, we analysed the results of all CRS and HIPEC treatments in the Netherlands with a uniform protocol considering timing and chemotherapy use.

## Patients and Methods

### Patient Selection

**Fig. 1:**
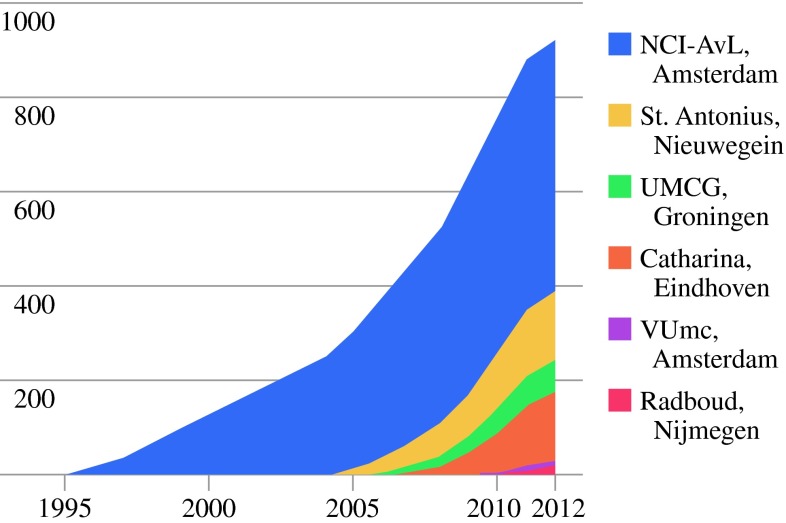
Cumulative number of patients who underwent CRS-HIPEC in the Netherlands over the years

All patients that underwent CRS–HIPEC for peritoneal disease from colorectal origin in the Netherlands were included. The peritoneal malignancies originated from colorectal cancer, appendiceal cancer, or pseudomyxoma peritonei (PMP). Patients were included between November 1995 and June 2012. The different institutions started at different time points (Fig. 1).

### Treatment

The institutions performed the CRS–HIPEC procedure under the same standardized protocol. Extensive debulking with peritonectomy and, when needed, multiorgan resections were performed, as described by Sugarbaker et al.^10^,^11^ and all the latter recommendations. The purpose of the cytoreduction was to obtain a macroscopically complete CRS (R1) resection, which means that no macroscopically visible residual tumor was left at the end of the surgical resection. After the cytoreduction, the open perfusion protocol of the abdominal cavity with mitomycin C was performed.^17^ The inflow temperature of the perfusate was 41–42 °C. As soon as this temperature was reached, mitomycin C was added, 35 mg/m^2^ body surface, in three fractions (one half, one fourth, and one fourth of the total dose) with a 30-min interval. Mitomycin C was used under the same schedule for all first HIPEC procedures. If a patient had undergone a HIPEC before, procedures were done with intraperitoneal oxaliplatin (460 mg/m^2^), systemic folinic acid (20 mg/m^2^), and 5-fluorouracil (5-FU; 400 mg/m^2^). When new institutions started performing CRS–HIPEC, a surgeon of an experienced institute monitored the procedure to ensure that the procedure was performed according to the Dutch HIPEC protocol.

### Data Collection and Analysis

Prospective databases and medical charts were reviewed for patient’s characteristics, operative details, pathology reports, and outcome. In colorectal carcinoma, histological mucinous adenocarcinoma was defined as carcinoma with 50 % of mucus. If there were any signet ring cells found in the tumour, it was considered a signet ring cell carcinoma. The participating hospitals used different classification methods for PMP. The classification system first described by Ronnett et al.^18^ that classifies PMP in DPAM, PMCA, and PMCA-I was used. Also, the more recent classification in low (DPAM) and high (PMCA/I) grade PMP was used, presented by the WHO in 2010.^19^ For this study, the latter grading system was applied or converted into. Tumour load was measured by counting the affected abdominal regions (0–7).^20^

Completeness of CRS was determined according to the maximum thickness of tumor nodules left behind. No residual macroscopic tumor was graded as an R1 resection, residual macroscopic tumor <2.5 mm was recorded as an R2a resection and if more disease was left behind, this was graded as an R2b resection.^13^ Hospital stay was defined as time in days from date of surgery to discharge. Morbidity was graded by the National Cancer Institute’s Common Toxicity Criteria of Adverse Events version 4.0 (CTCAE v4.0).^21^ For every procedure, the complication with the highest grade was listed.

Survival was analysed separately for colorectal cancer and PMP patients because of the different course of the disease. Progression-free survival was measured from the date of the HIPEC procedure until date of progression of the disease or date of last follow-up in censored cases. Overall survival was measured by the date of the HIPEC procedure and date of death or last follow-up date. Median progression free and overall survivals were expressed in months. Furthermore, 3- and 5-year overall survival rates were measured.

### Statistical Analysis

Clinicopathological characteristics and surgical outcome were analyzed by descriptive statistics. Categorical variables were compared using Chi-square analysis or Fisher’s exact test where appropriate. Normally distributed variables were compared using the *t* test or one-way ANOVA as appropriate, nonparametric tests were used when variables were not normally distributed. Survival was measured with the Kaplan–Meier method. *p* < 0.05 was considered significant in all analysis. Survival analysis was performed under intention-to-treat conditions, which means that patients with incomplete resections were included. All statistical analyses were conducted using SPSS software (version 20, SPSS Inc., Chicago, IL, USA).

## Results

Clinicopathological characteristics are presented in Table 1; 960 patients were included in the study, and 660 patients were affected by PC of colorectal cancer (69 %) and the remaining suffered from PMP (31 %). Median age at the time of the CRS and HIPEC procedure was 58 years for both CRC (range 21–79 years) and PMP (range 28–81 years; *p* = 0.271); 386 patients (40 %) were male. the Netherlands Cancer Institute included 554 patients until 2012, St. Antonius Hospital Nieuwegein 151 patients, University Medical Centre Groningen 72 patients, Catharina Hospital Eindhoven 151 patients, Radboud University Nijmegen Medical Center 13 patients, and VU medical centre 19 patients. The total number of CRS and HIPEC procedures per year increased from 21 in 1995 to 136 procedures in 2011. The cumulative number of patients included in this study over time is shown in Fig. 1.

**Table 1:** Clinicopathological characteristics

	CRC		PMP		*p* value
Characteristic	No. of patients		No. of patients		
	*n* = 660		*n* = 300		
Age (year)					
Median (range)	58 (21–79)		58 (28–81)		0.271^b^
Gender					
Male	297	45 %	89	30 %	<0.001^c^
Female	363	55 %	210	70 %	
Hospital					
1	334	51 %	220	73 %	
2	121	18 %	30	10 %	
3	48	7 %	24	8 %	
4	128	19 %	23	8 %	
5	12	2 %	1	0.3 %	
6	17	3 %	2	0.7 %	
Year of surgery					
1995–1999	61	12/year	44	9/year	
2000–2004	89	18/year	69	14/year	
2005–2009	274	55/year	116	23/year	
2010	101	101/year	27	27/year	
2011	102	102/year	34	34/year	
2012^a^	33		10		
Primary localisation					
Appendix	62	9 %			
Right colon	193	29 %			
Transverse colon	30	5 %			
Left colon	51	8 %			
Rectosigmoid	308	47 %			
Unknown	16	2 %	43	14 %	
Histology	24	4 %			
Intestinal type					
Signet cell ring	556	84 %			
Mucinous	24	4 %			
Low grade	70	13 %	140	47 %	
High grade			49	16 %	
Unknown			111	37 %	
Tumour differentiation					
Well	36	6 %			
Moderately	203	37 %			
Poor	70	13 %			
Unknown	247	44 %			
Lymph node involvement					
Positive	365	55 %			
Negative	192	29 %			
Unknown	103	16 %			
Synchronous PC					
Yes	299	45 %			
No	269	41 %			
Unknown	92	14 %			
Abdominal region involvement					
Median (range)	3 (1–7)		5 (1–7)		<0.001^b^

*CRC* colorectal carcinoma, *PMP* pseudomyxoma peritonei

*1* Netherlands Cancer Institute—Antoni van Leeuwenhoek, Amsterdam, *2* Antonius Hospital Nieuwegein, *3* University Medical Centre Groningen, *4* Catharina Hospital Eindhoven, *5* Radboud University Nijmegen Medical Center, *6* VU medical centre, Amsterdam

^a^Patients were included to 2012 where the data were available

^b^Mann–Whitney *U* test

^c^Chi-square test

The primary tumour of the colorectal cancer patients was located in the appendix (9 %), right colon (29 %), transverse colon (5 %), left colon (8 %), and rectosigmoid (47 %). Most of the patients had intestinal-type adenocarcinoma (84 %); a lower number of patients had mucinous adenocarcinoma (12 %) or signet ring cell carcinoma (4 %). Tumour differentiation was mentioned in the pathology report in 56 % of the cases. Six percent of tumours were well differentiated, 37 % were moderately differentiated, and 13 % were poorly differentiated. Lymph nodes were involved at the time of the primary resection in 55 % of the patients. Synchronous peritoneal metastases were found in 45 % of the patients.

Patients with PMP had a primary neoplasm in the appendix in 86 % of the cases. The other 14 % were found in the caecal region, elsewhere in the colon, or in the ovaries. In little cases, no primary lesion was found. Not all pathology reports (37 %) mentioned subclassification of PMP. Forty-seven percent of the patients had low-grade PMP and 16 % had high-grade PMP. Median abdominal regions affected were 3 (range 1–7) regions for the CRC patients and 5 (range 1–7) regions for the PMP patients (*p* < 0.001).

Surgical outcome and morbidity are presented in Table 2. In 767 patients (80 %), an R1 cytoreduction was achieved. Major complications, grade III–V, occurred in 331 (34 %) procedures. Thirty-two patients died from a complicated procedure; consequently the mortality rate was 3 %. The most common cause of mortality was anastomotic leakage. Median hospital stay was 16 days (95 % confidence interval (CI) 13–22 days).

**Table 2:** Surgical outcome

Outcome	No. of patients *n* = 960	%
Cytoreduction		
R1	767	80
R2a/b	187	19
Unknown	6	1
Major complication		
Yes	331	34
No	581	61
Unknown	48	5
Mortality		
Yes	32	3
Anastomotic leakage	11	97
Bowel perforation	5	
Respiratory insufficiency	4	
Fatal haemorrhage	3	
Fistula	2	
Bile leakage	1	
Cardiac arrest	1	
Infection	1	
Pulmonary embolism	1	
Necrotic bowel	1	
Pancreatitis	1	
Cerebral vascular accident	1	
No	928	
Hospital stay	16 (0–166)	
Days, median (range)		

*R1* no macroscopic residual disease, *R2a/b* macroscopic residual disease

**Fig. 2:**
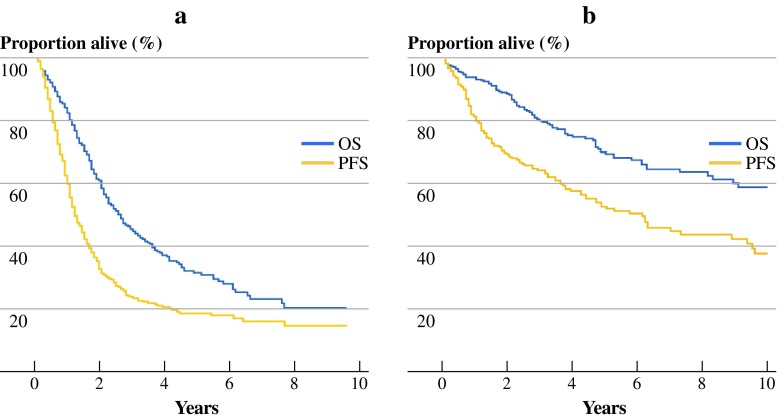
**a** Survival of patients with PC of colorectal cancer (*n* = 660) in Kaplan–Meier survival curve. **b** Survival of patients with PMP (*n* = 300) in Kaplan–Meier survival curve. *OS* overall survival, *PFS* progression-free survival

Survival was analysed separately for peritoneal metastasis of colorectal carcinoma and PMP (Table 3); 401 patients were deceased (42 %) at the time of analysis. Median follow-up time was 41 months (95 % CI 36–46 months). Median progression-free survival (PFS) for colorectal carcinoma patients was 15 months (95 % CI 13–17 months; Fig. 2a). For PMP patients, median progression-free survival was 53 months (95 % CI 40–60 months; Fig. 2b). Median overall survival (OS) for colorectal carcinoma patients was 33 months (95 % CI 28–38 months). The 3-year survival rate was 46 %, and the 5-year survival rate was 31 % (Fig. 2a). For PMP patients the median OS was 130 months (95 % CI 98–162 months). The 3-year survival rate for PMP patients was 77 % and the 5-year survival rate was 65 % (Fig. 2b).

**Table 3:** Survival

	PC of CRC	PMP
	*n* = 660	*n* = 300
PFS		
Median (95 % CI)	15 (13–17)	53 (40–66)
OS		
Median (95 % CI)	33 (28–38)	130 (98–162)
3-year	46 %	77 %
5-year	31 %	65 %

*PFS* progression-free survival in months, *OS* overall survival in months, *CI* confidence interval

## Discussion

Since the first publication of Sugarbaker on CRS–HIPEC as a possible curative treatment for peritoneal surface malignancies, many institutions started to perform this procedure.^10^ All centres in the Netherlands agreed on monitoring safety of the CRS and HIPEC and adopted the uniform Dutch HIPEC protocol. The combined results of all centres showed a median survival of 33 months (95 % CI 28–38 months) for PC of colorectal cancer and 130 months (95 % CI 98–162 months) for patients with PMP.

Compared with the results of the randomized trial from Verwaal et al.^1^,^14^ the proportion of complete cytoreductions has increased from 37 to 80 %. Major complication and mortality rates were 34 and 3 % in this study, which is comparable to the literature, but lower than in the randomized trial. Even though patient selection has become more cautious over the years, these results still suggest that broader implementation of the treatment at least did not have a negative consequence for the surgical outcome.

Survival rates for both PC from colorectal carcinoma and PMP were comparable to literature. A number of CRS–HIPEC studies have been published with survival numbers for the different peritoneal surface malignancies. Most of these studies are limited in numbers. The study with the largest number that has recently been published is the French multi-institutional study by Glehen et al.^14^ In this study, 1,290 patients were included. Median follow-up was 45.3 months. Median overall survival was 30 months for colorectal cancer and for PMP was not yet reached. Three- and five-year survival was 41 and 26 % respectively for colorectal carcinoma and 85 and 73 % for PMP. Twenty-five institutions participated in the study with different levels of expertise and with different treatment regimens. In 86 % of the cases, HIPEC was performed either with mitomycin C or oxaliplatin. EPIC instead of HIPEC was performed in 14 % of the cases. The results of the present study are roughly comparable to the results of the French study. In both studies, a leading HIPEC centre might have had a strong influence on the outcome. However, there is a major difference in the wider use of the CRS and HIPEC treatment. In the French study, the treatment protocols differ between the hospitals, so the results reflect the mean outcome of different CRS–HIPEC regimens, while in the Netherlands there is a unique situation; the use of a national uniform treatment protocol.

Because HIPEC is only performed in specialised centres, there might be a certain patient selection. Some oncologists claim that patients that choose to undergo a HIPEC are generally younger, healthier, wealthier, and more sensitive to chemotherapy. These patients might have limited disease compared to patients who undergo chemotherapy.^22^ However, there is no evidence on this subject to confirm this clinical opinion. In this study, patients were treated in one of the six hospitals that perform CRS–HIPEC in the Netherlands. The centres that participated are different centres: academic, nonacademic, large periphery hospitals, and a specialized cancer centre. So, not only patients that are referred to tertiary referral hospitals are included in this study, but also patients that present with peritoneal malignancies in other hospitals. Inevitably, there is a certain patient selection for CRS–HIPEC. The treatment includes abdominal surgery, which has certain risks. Patients in a nonoptimal physical and mental condition with a large extent of disease do not benefit of the treatment. Still, many patients, older and younger, are in a good enough condition to undergo the treatment. Whether patients in HIPEC studies are more sensitive to chemotherapy remains to be discussed. It has been studied that administration of (neo) adjuvant chemotherapy is regularly a prognostic factor for survival after CRS-HIPEC, though the best timing and regimen are still unknown.^14^,^23^,^24^ The purpose for this study was to investigate whether the widespread availability of the CRS–HIPEC procedure to more hospitals would endanger the outcome, but this we could not confirm. Mentioned should be that patients in a poor health status and having bowel obstructions are not likely to be eligible for CRS–HIPEC, neither for systemic chemotherapy.

Furthermore, critics state that modern day chemotherapy has better results on metastatic colorectal cancer than the 5-fluorouracil based chemotherapy referred to in most available publications.^22^ Yet, no studies have been published that compare newest chemotherapy regimens with CRS–HIPEC. Peritoneal metastases are not always analysed separately in large metastatic colorectal cancer chemotherapy trials because of the difficulty in monitoring the peritoneal lesions. CT scans and PET scans do not pick up small peritoneal lesions easily, so response measurement is problematic. Analysis of large combination chemotherapy trials revealed that peritoneal carcinomatosis among patients with metastatic colorectal cancer is associated with a 30 % reduction in overall survival (10.7 vs. 17.6 months). These numbers are not fully comparable to CRS–HIPEC studies, as the patients in this study regularly have more metastatic sites, such as liver or lung metastases.^8^ In recent years, chemotherapy development has progressed. There are indications that targeted therapies can be useful to treat peritoneal metastasis from colorectal cancer.^9^ In the CAIRO2 study, median survival for conventional therapy, capecitabine with oxaliplatin and bevacizumab, was 15.2 and 13.9 months for patients for which cetuximab was added to the regimen. In future studies, it would be interesting to investigate the benefit of targeted therapies for patients with peritoneal metastases, alone or in combination with CRS–HIPEC. Recently, the COMBATAC trial has started to investigate the benefit of adding targeted therapy to the CRS–HIPEC treatment.^25^

This study is limited by the multicentre design. Moreover, a disproportionate amount of patients was treated in the Netherlands Cancer Institute (51 % of colorectal cancer patients and 73 % of PMP patients) because of their longer experience with the CRS–HIPEC procedure (Table 1). The experience of the hospitals on CRS–HIPEC differed, but they all used the Dutch HIPEC protocol. Surgical outcome appeared to be similar between the hospitals (unpublished data).

This study shows that, following the randomized trial, the Dutch protocol is a safe approach for widespread use of the CRS–HIPEC treatment with tolerable morbidity and convincing survival. It is important that more research is performed to find better chemotherapy combined with CRS–HIPEC for patients with peritoneal disease of colorectal origin. Until then, CRS–HIPEC provides the best chance of survival.
